# Intrapulmonary and Intracardiac Shunts in Adult COVID-19 Versus Non-COVID Acute Respiratory Distress Syndrome ICU Patients Using Echocardiography and Contrast Bubble Studies (COVID-Shunt Study): A Prospective, Observational Cohort Study

**DOI:** 10.1097/CCM.0000000000005848

**Published:** 2023-03-27

**Authors:** Vincent I. Lau, Graham D. Mah, Xiaoming Wang, Leon Byker, Andrea Robinson, Lazar Milovanovic, Aws Alherbish, Jeffrey Odenbach, Cristian Vadeanu, David Lu, Leo Smyth, Mitchell Rohatensky, Brian Whiteside, Phillip Gregoire, Warren Luksun, Sean van Diepen, Dustin Anderson, Sanam Verma, Jocelyn Slemko, Peter Brindley, Demetrios J. Kustogiannis, Michael Jacka, Andrew Shaw, Matt Wheatley, Jonathan Windram, Dawn Opgenorth, Nadia Baig, Oleksa G. Rewa, Sean M. Bagshaw, Brian M. Buchanan

**Affiliations:** 1 Department of Critical Care Medicine, Faculty of Medicine and Dentistry, University of Alberta, and Alberta Health Services, Edmonton, AB, Canada.; 2 Health Services Statistical and Analytic Methods, Alberta Health Services, Edmonton, AB, Canada.; 3 Division of Cardiology, Department of Medicine, Faculty of Medicine, and Alberta Health Services, Edmonton, AB, Canada.; 4 Department of Critical Care Medicine, Cumming School of Medicine, University of Calgary, Calgary, AB, Canada.; 5 Department of Emergency Medicine, Faculty of Medicine and Dentistry, University of Alberta, Edmonton, AB, Canada.; 6 Faculty of Medicine and Dentistry, University of Alberta, Edmonton, AB, Canada.; 7 Department of Medicine, Cumming School of Medicine, University of Calgary, Calgary, AB, Canada.; 8 Department of Anesthesiology & Pain Medicine, Faculty of Medicine and Dentistry, University of Alberta, Edmonton, AB, Canada.; 9 Division of Neurology, Department of Medicine, Faculty of Medicine and Dentistry, University of Alberta, Edmonton, AB, Canada.; 10 Department of Intensive Care and Resuscitation, Cleveland Clinic, Cleveland, OH.; 11 Department of Neurosurgery, Faculty of Medicine and Dentistry, University of Alberta, Edmonton, AB, Canada.; 12 School of Public Health, University of Alberta, Edmonton, AB, Canada.

**Keywords:** bubble study, COVID-19, echocardiography, hypoxemia, shunt, transcranial Doppler

## Abstract

Studies have suggested intrapulmonary shunts may contribute to hypoxemia in COVID-19 acute respiratory distress syndrome (ARDS) with worse associated outcomes. We evaluated the presence of right-to-left (R-L) shunts in COVID-19 and non-COVID ARDS patients using a comprehensive hypoxemia workup for shunt etiology and associations with mortality.

**DESIGN:**

Prospective, observational cohort study.

**SETTING:**

Four tertiary hospitals in Edmonton, Alberta, Canada.

**PATIENTS:**

Adult critically ill, mechanically ventilated, ICU patients admitted with COVID-19 or non-COVID (November 16, 2020, to September 1, 2021).

**INTERVENTIONS:**

Agitated-saline bubble studies with transthoracic echocardiography/transcranial Doppler ± transesophageal echocardiography assessed for R-L shunts presence.

**MEASUREMENTS AND MAIN RESULTS:**

Primary outcomes were shunt frequency and association with hospital mortality. Logistic regression analysis was used for adjustment. The study enrolled 226 patients (182 COVID-19 vs 42 non-COVID). Median age was 58 years (interquartile range [IQR], 47–67 yr) and Acute Physiology and Chronic Health Evaluation II scores of 30 (IQR, 21–36). In COVID-19 patients, the frequency of R-L shunt was 31 of 182 COVID patients (17.0%) versus 10 of 44 non-COVID patients (22.7%), with no difference detected in shunt rates (risk difference [RD], –5.7%; 95% CI, –18.4 to 7.0; *p* = 0.38). In the COVID-19 group, hospital mortality was higher for those with R-L shunt compared with those without (54.8% vs 35.8%; RD, 19.0%; 95% CI, 0.1–37.9; *p* = 0.05). This did not persist at 90-day mortality nor after adjustment with regression.

**CONCLUSIONS:**

There was no evidence of increased R-L shunt rates in COVID-19 compared with non-COVID controls. R-L shunt was associated with increased in-hospital mortality for COVID-19 patients, but this did not persist at 90-day mortality or after adjusting using logistic regression.

**KEY POINTS**

**Question:** Does right-to-left (R-L) shunt frequency increase with COVID-19 acute respiratory distress syndrome (ARDS) compared with non-COVID patients, and is there association with shunt frequency and mortality?

**Findings:** There was no difference in shunt frequency between COVID-19 and non-COVID ARDS patients. In COVID-19 patients, there was increased hospital mortality for those with shunt versus those without, but this difference did not persist at 90-day mortality nor after logistic regression adjustment.

**Meaning:** COVID-19 does not increase shunt frequency as compared with non-COVID or historical controls. R-L shunt presence may be associated with increased hospital mortality for COVID-19 patients but not at 90 days or after regression adjustment.

Severe acute respiratory syndrome coronavirus 2, or COVID-19, has infected at least 500 million people and killed over 6 million. The primary cause of death is usually intractable hypoxemia from acute respiratory distress syndrome (ARDS) (1). However, some literature raised the possibility of other causes of hypoxemia: specifically, right-to-left (R-L) shunts (2). Autopsies from COVID-19 pneumonia patients also demonstrated pulmonary capillary deformations (3), and dual-energy CT images suggested pulmonary vessel dilatation (4).

A recent study reported a R-L shunt in 83% of adult ICU patients with severe COVID-19 (5). The authors concluded this was secondary to increased pulmonary vascular dilation. However, sample size was small (*n* = 18), and they relied upon agitated-saline microbubbles via transcranial Doppler (TCD) of the bilateral middle cerebral arteries (6). However, they could not rule out intracardiac disease, as neither transthoracic echocardiography (TTE) nor transesophageal echocardiography (TEE) was performed (6). This frequency of shunt was significantly higher than historical ARDS controls (5, 7), it raised the possibility that COVID-19 ARDS might be associated with increased R-L shunt.In contrast, another study reported lower rates of shunt in COVID-19 ARDS patients: 10% with patent foramen ovale (PFO) and 20% with detectable transpulmonary bubble transit (8), more in-line with historical controls (7, 9). However, numbers were relatively low (*n* = 60) and the study used contrast-enhanced TTE (but not TEE). While an improvement on TCD, TTE lacks sufficient sensitivity to fully assess the intra-atrial septum (8, 10, 11).

A recent systematic review suggested an association between R-L shunts and increased mortality (12). Therefore, the purpose of our study was to compare COVID-19 ARDS and non-COVID-19 ARDS ICU patients for R-L shunt presence, shunt etiology (intrapulmonary/intracardiac), and associations with mortality. We used a comprehensive hypoxemia protocol that included contrast-enhanced TTE/TCD and TEE.

## METHODS

This study was reviewed and fully approved by the local institutional review board (University of Alberta Research Ethics Board: PRO00104364, approved October 20, 2020), with procedures followed in accordance with the ethical standards of the responsible committee on human experimentation (institutional or regional) and with the Helsinki Declaration of 1975. Waived consent for data was obtained given that TTE, TCD, and TEE are all within standard of care for severe hypoxemia at our institution (and de-identified registry data was available for all patients). Clinical assent/consent for TEE was obtained from either the patient’s substitute decision-maker and/or attending physician.

### Setting and Study Design

Four Canadian ICUs in Edmonton, Alberta, Canada, participated in this prospective, observational cohort study. All are tertiary care referral centers, caring for complex medical, trauma, surgical, oncologic, and transplant patients. All sites are equipped with portable ultrasound machines (Fujifilm Sonosite, Bothell, WA) with probes for TTE, TEE, and TCD. Following a point-of-care ultrasound (POCUS) study by the physician, images are saved and automatically uploaded to the Qpath (Telexy, Maple Ridge, BC, Canada) archiving system, along with a report charted from the scanning physician.

We recruited eligible all consecutive ARDS COVID and non-COVID patients between November 16, 2020, and September 1, 2021, who all received a protocolized hypoxemia shunt workup. Patients were included if they were diagnosed with ARDS who were receiving invasive mechanical ventilation plus COVID-19 pneumonia (comparator group) versus ARDS without COVID (control group). Patients were excluded if less than 18 years old.

### Working Definitions

We defined COVID-19 infection as having a polymerase chain reaction nucleic acid test confirmed by healthcare-approved assay (reference).

We used the 2012 Berlin ARDS definition (13). We defined and diagnosed R-L intrapulmonary shunts and intracardiac shunts as per the American Society of Echocardiography, where intracardiac shunt was defined as a positive bubble study usually within 1–2 cardiac cycles, and evidence of PFO/atrial septal defect (ASD) via TTE or TEE with color Doppler (11, 14). An intrapulmonary shunt was defined as evidence of positive bubble study usually within 4–8 cardiac cycles, with no evidence of PFO/ASD on a TTE or TEE with color Doppler (11, 14).

A positive TCD study was defined by detection of any microbubbles during insonation of the middle cerebral artery with pulse-wave Doppler and injection of agitated-saline contrast with and without simulated Valsalva (simulating increased intra-abdominal pressure by pressing on the abdomen and then releasing). We did not categorize severity, only the binary presence/absence of a R-L shunt by TCD (15).

### Hypoxemia Shunt Workup

We performed an intracardiac and intrapulmonary shunt workup for hypoxemia in COVID-19 and non-COVID ARDS patients. These studies were performed within 72 hours of initiation of mechanical ventilation and ICU admission and were typically performed in under 24 hours. All components of the workup (TTE/TCD/TEE) were performed within the same day. All operators and sonographers wore full personal protective equipment. The shunt bubble study protocol is further outlined in **Supplemental Figure 1** (http://links.lww.com/CCM/H312), including full explanations of TTE/TCD/TEE protocols.

All patient investigations adhered to American Society of Echocardiography (11, 14) or American Society of Neuroimaging standards (15, 16). All studies were supervised by board-certified echocardiographers or TCD sonographers from critical care/cardiac anesthesia/cardiology physicians. We performed external validation with over-readers of our TTE/TEE/TCD bubble studies, which allowed us to calculate inter-rater reliability (kappa statistic) to examine agreement in diagnosis of findings.

### Data Collection

The Qpath database was queried for all TTE/TCD/TEE images/clips and reports. Demographic and clinical characteristic data were collected from registry databases within Alberta Health Services (eCritical/data warehouse and clinical analytics system/Data Integration, Measurement & Reporting) and included: COVID-19 status, age, sex, race/ethnicity, patient case-mix (medical, surgical, trauma), Acute Physiology And Chronic Health Evaluation (APACHE) II score (17), Charlson Comorbidity Index (CCI) score (18), respiratory mechanics (e.g., tidal volumes [TVs], positive end-expiratory pressure [PEEP] static compliance, plateau pressures, Pao_2_/Fio_2_ [PF] ratio, arterial blood gas results [including alveolar-arterial gradient]), deadspace calculations (using arterial blood gas Pco_2_ compared with end-tidal Co_2_ from volumetric capnography), and type of ventilation at time of bubble study, types of interventions during hospital stay (e.g., prone positioning, airway-pressure release ventilation, pulmonary vasodilators, extracorporeal membrane oxygenation, renal replacement therapy), vasopressors/inotropes, steroid use, stress ulcer prophylaxis, venous thromboembolism prophylaxis, ventilator-associated pneumonia prophylaxis, sedation, analgesia, neuromuscular blockade use, and other baseline measures (e.g., vitals signs, laboratory values: complete blood count, troponin, d-dimer), where available. All laboratories were collected as part of the clinical team’s discretion, and we did not require brain natriuretic peptide collection as per study protocols.

The echocardiographic findings collected were: date of study, POCUS examination type (TTE or TEE) and location, presence/absence of intracardiac versus intrapulmonary shunt by bubble study, presence/absence of intra-atrial septal defect by color Doppler, and all other echocardiographic findings: for example, biventricular size and function, valvulopathy, pericardial disease, superior or inferior vena caval size, and respirophasic changes, etc. Tricuspid annular planar systolic excursion (TAPSE) and pulmonary acceleration time (PAT) and shunt fractions were not routinely measured for all studies due to variations in technically challenging patient anatomy and image acquisition skills of sonographers. All findings were overread for quality assurance (e.g., right ventricular [RV] size and dysfunction assessment was performed using eye-ball estimation of function; and, the absence of tricuspid regurgitation did not exclude the presence of pulmonary hypertension but only that there might have been insufficient tricuspid regurgitation to quantify the degree of pulmonary hypertension), and all study images were reviewed by at least two expert echocardiographers with National Board of Echocardiography certification to calculate inter-rater reliability for shunt identification. These POCUS assessors were blinded to clinical outcomes during the quality assurance oversight process. Treatment teams were not blinded to POCUS findings.

Clinical outcomes were reported through hospital discharge and at 90 days post-ICU admission. These included: ICU length of stay, hospital length of stay, duration of mechanical ventilation, and complications related to study procedures and hospitalization (**Supplemental Appendix 1**, http://links.lww.com/CCM/H312).

### Statistical Analysis and Sample Size

Descriptive statistics were generated for baseline demographic, clinical characteristics, echocardiographic findings, and clinical outcome variables. Categorical data were summarized using frequency and column percentage and normal distributed data were described using mean and sd. Non-normal distributed data were presented as median and interquartile ranges (IQRs). Data were compared (where appropriate) using a Pearson chi-square test (categorical data), Student *t* test (normal distributed data), and nonparametric Kruskal-Wallis test (non-normal distributed data). A *p* value of less than 0.05 was considered statistically significant with 95% CIs also reported, if applicable. Missing data or lost-to-follow-up was less than 5%, so no imputation was required. There were no prespecified sensitivity analyses or subgroups.

Inter-rater reliability for echocardiographic findings of shunt (intrapulmonary vs intracardiac) was calculated for Cohen’s kappa statistic, where the following interpretations were used: less than 0 (poor), 0–0.20 (slight), 0.21–0.40 (fair), 0.41–0.60 (moderate), 0.61–0.80 (substantial), and 0.81–1.00 (almost perfect) (19, 20).

Univariate logistic regression modeling was used to evaluate the association between unadjusted odds ratios (ORs) with 95% CIs with mortality (continuous). Multivariable logistic regression modeling was also used to calculate adjusted ORs, adjusting for known variables including baseline demographics (age, sex) and clinical characteristics (CCI) and (illness severity scores: e.g., APACHE II) to determine if the presence of shunt mortality exists after adjustment. These variables were prespecified a priori. Given prior evidence of lack of association between PF ratios and shunt presence (12), this was not a variable used for adjustment.

These statistical analyses were performed using Statistical Analysis System Enterprise Guide 7.1 (Cary, NC) or Microsoft Excel, Version 14.0.6 (Microsoft Corporation, Redmond, WA). All reporting of this observational cohort study was made in accordance with the Strengthening the Reporting of Observational Studies in Epidemiology (STROBE) guidelines and checklist (**Supplemental Appendix 2**, http://links.lww.com/CCM/H312) (21).

In order to calculate study power, we used a reported frequency of shunts (e.g., PFOs) of approximately 19% in severe pneumonia/ARDS and a predicted increase in shunt of 15% with cor pulmonale (right-sided heart failure) physiology (up to a shunt rate of 34%) (7). Using an alpha of 0.05 and power of 0.80, we calculated a minimum total sample size of 212 patients (106 patients per group). Considering an approximate attrition rate of 5%, this would require a minimum of 224 study participants for the incident shunt rate in the study.

## RESULTS

### Demographics and Clinical Characteristics

**Figure 1.**
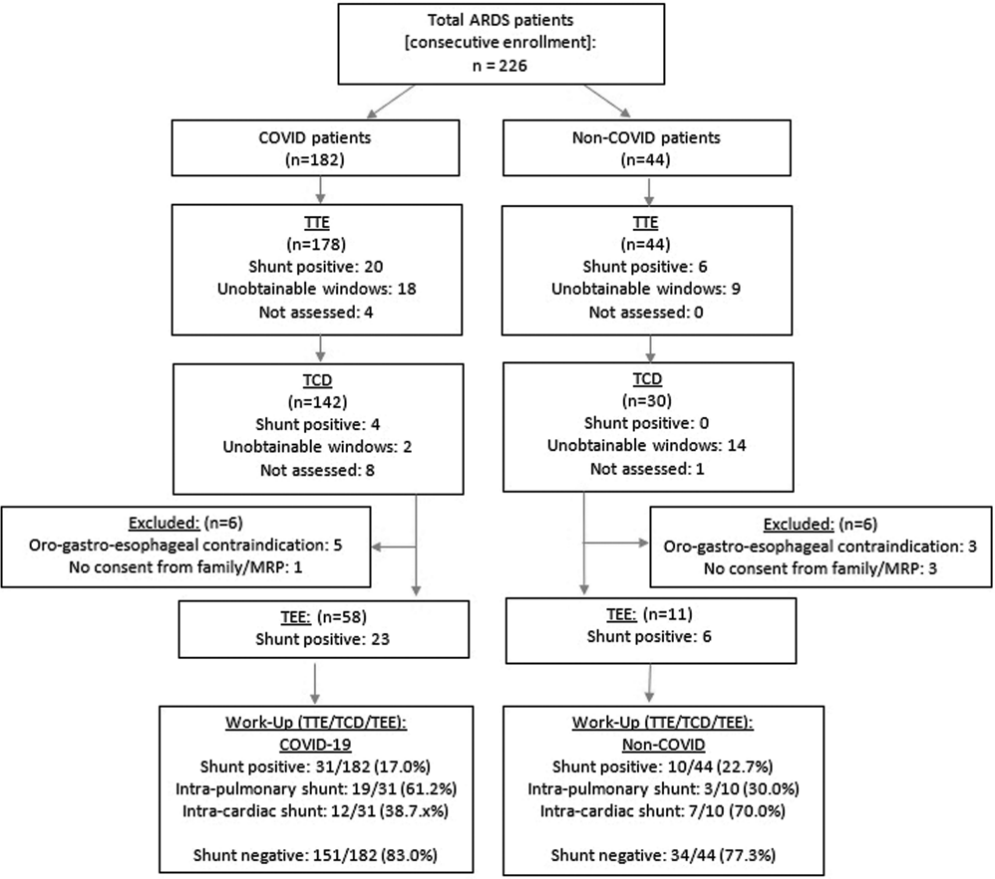
Strengthening the Reporting of Observational Studies in Epidemiology diagram flowchart for hypoxemia workup assessing shunt presence. ARDS = acute respiratory distress syndrome, MRP = most responsible physician, TCD = transcranial Doppler, TEE = transesophageal echocardiography, TTE = transthoracic echocardiography.

We enrolled 226 patients. Of these, 182 were COVID-19 positive, and 44 composed the non-COVID control group (**Fig. 1**). Baseline demographics and clinical characteristics are presented in **Supplemental Table 1** (http://links.lww.com/CCM/H312). Both groups had comparable TVs, plateau pressures, and static compliance in keeping with high and equivalent rates of lung protective ventilation in both groups. The COVID-19 arm was associated with significantly higher rates of noninvasive positive pressure ventilation and high-flow nasal cannula oxygen administration (92.9% vs 77.3%; risk difference [RD], 15.6%; 95% CI, 5.7–25.5; *p* = 0.001) prior to intubation (Supplemental Table 1, http://links.lww.com/CCM/H312). More patients in the COVID-19 arm underwent prone positioning (77.5% vs 43.2%; RD, 34.3%; 95% CI, 19.3–49.3; *p* = 0.000004) (**Supplemental Table 2**, http://links.lww.com/CCM/H312). Percent positivity of R-L started at ~27% in the beginning of the study but fell to ~17% by the end (**Supplemental Fig. 2**, http://links.lww.com/CCM/H312). There were two patients with preexisting shunts in the non-COVID groups (2/34 patients, 5.9%), which were later classified in the nonshunt group, as they had negative R-L bubble studies and no visible shunts on color Doppler (Supplemental Table 1, http://links.lww.com/CCM/H312).

### Echocardiographic Findings, R-L Shunts, and Inter-Rater Reliability

Echocardiographic findings and the percentage with a R-L shunt are shown in **Supplemental Table 3** (http://links.lww.com/CCM/H312). In the COVID-19 group, 31 of 182 patients (17.0%) had a shunt identified, of which 12 were intracardiac (38.7%) and 19 (61.3%) were intrapulmonary shunts. In the non-COVID group, 10 of 44 patients (22.7%) had an identified shunt; of which 7 (70.0%) were intracardiac shunts and 3 (30.0%) were intrapulmonary. There was no statistically significant difference in the overall rate of shunt between the COVID-19 and non-COVID groups (17.0% vs 22.7%; RD, –5.7%; 95% CI, –18.4 to 7.0; *p* = 0.38). There was a nonsignificant higher proportion of intrapulmonary shunts in the COVID-19 group compared with non-COVID (61.2% vs 30.0%, respectively; RD, 31.2%; 95% CI, –4.4% to 66.8%; *p* = 0.08) (Supplemental Table 3, http://links.lww.com/CCM/H312). Inter-rater reliability was high for TTE/TCD/TEE shown in **Supplemental Table 4** (http://links.lww.com/CCM/H312).

### Clinical Outcomes

Clinical outcomes are summarized in Supplemental Table 2 (http://links.lww.com/CCM/H312). For the primary outcome, there was higher in-hospital mortality among COVID shunt patients compared with no shunt (54.8% vs 35.8%; RD, 19.0%; 95% CI, 0.1–37.9; *p* = 0.05). However, this difference was no longer significant at 90-day mortality (54.8% vs 38.4%; RD, 16.4%; 95% CI, –2.6% to 35.4%; *p* = 0.10). There was no difference in either in-hospital (39.0% vs 43.2%; RD, –4.2%; 95% CI, –20.3% to 11.9%; *p* = 0.30) or 90-day mortality (41.2% vs 45.5%; RD, –4.1%; 95% CI, –12.2% to 20.4%; *p* = 0.31) between the COVID-19 and non-COVID arms.

COVID-19 infection was associated with a significantly longer median duration of mechanical ventilation (15.0 d [IQR, 8.0–25.0 d] vs 9.0 d [IQR, 5.0–17.0 d]; RD, 7.5 d; 95% CI, 0.5–14.5 d; *p* = 0.007) compared with the control group. There was also a longer median ICU length of stay (17.5 d [IQR, 11.0–28.0 d] vs 12.0 d [IQR, 7.5–20.0 d]; RD, 8.2 d; 95% CI, 1.0–15.4 d; *p* = 0.007).

There was no measurable increase in complications attributable to performing TEE (oropharyngeal/gastrointestinal bleeding or pneumomediastinum). There were no detected cases of esophageal perforation (Supplemental Table 2, http://links.lww.com/CCM/H312).

### Kaplan-Meier Curves and Multivariable Logistic Regression Analysis

**Figure 2.**
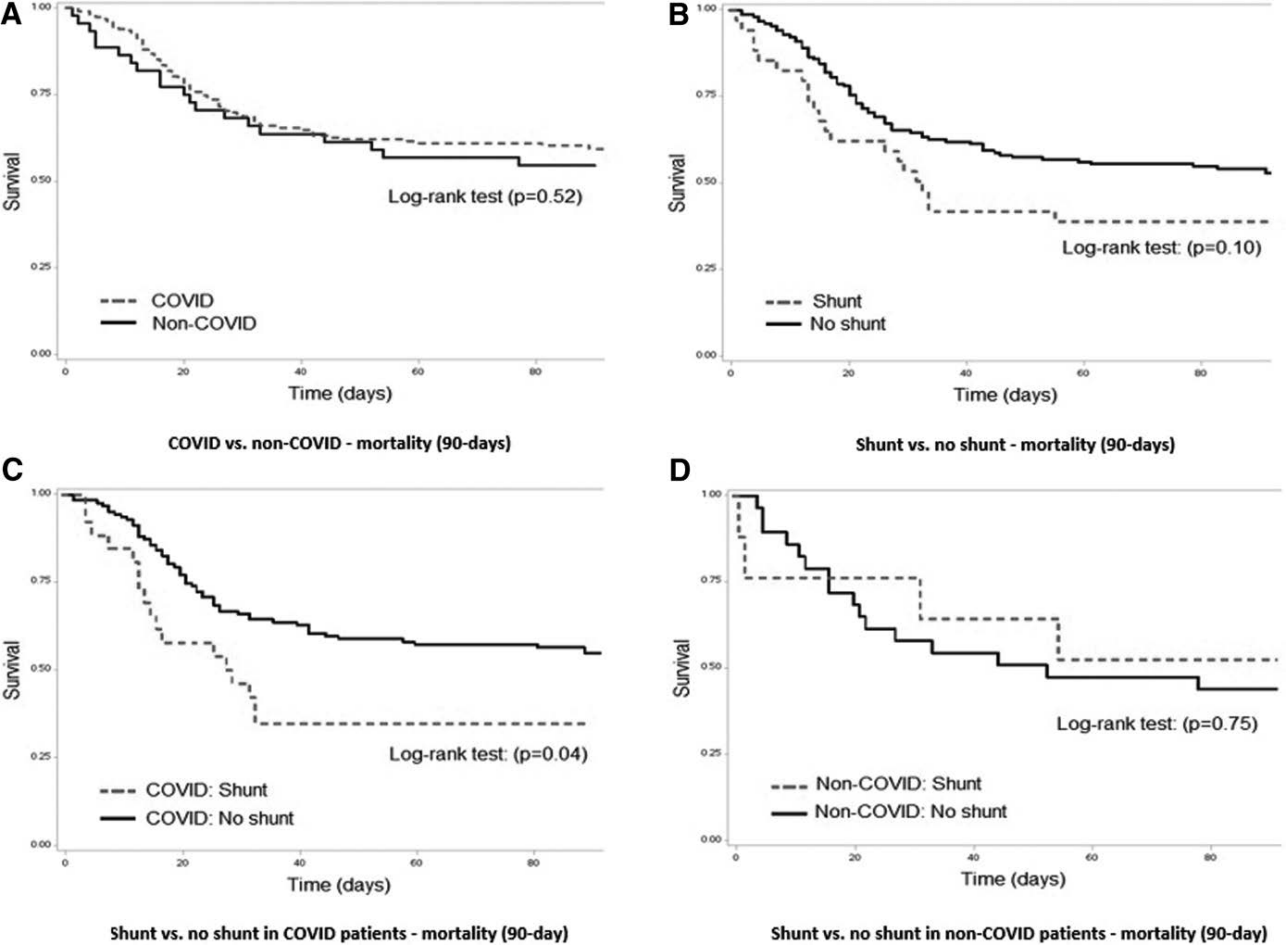
Kaplan-Meier curves.

Kaplan-Meier curves for 90-day mortality are presented in **Figure 2**. There was a significant difference in shunt versus no shunt in COVID patients (*p* = 0.04) (Fig. 2*C*). The remaining log-rank tests were not significant and after adjustment for multiple comparisons.

The combined regression analysis in the full cohort showed no significant difference in 90-day mortality based on the presence of any shunt or in the intracardiac and intrapulmonary shunt subtypes. The regression adjusting for Charlson’s Health Score specifically did show a significant increase in 90-day mortality in the shunt portion of the overall cohort (OR, 1.28; 95% CI, 1.09–1.52). No other individual covariable adjustments showed a significant signal for increased mortality in any group (**Supplemental Table 5**, http://links.lww.com/CCM/H312).

## DISCUSSION

In this study, COVID-19 shunt rates were not significantly different compared with non-COVID ARDS. Our findings align with our recent meta-analysis (12), suggesting approximately one in five patients with ARDS had a R-L shunt. Although there was no statistical significance for intracardiac or intrapulmonary shunt types between groups, there was a signal of higher intrapulmonary shunts in COVID patients, while higher intracardiac shunt rates in non-COVID patients, which may have been exacerbated by lower lung compliance with higher plateau pressures, leading to more intracardiac shunts in non-COVID patients. We also found an association between R-L shunts and increased hospital mortality, but this was no longer significant at 90-day mortality, or after multivariable adjustment.

Given that approximately one-in-five patients with ARDS may have a R-L shunt, this study is a reminder to clinicians to consider screening patients and, if present, to consider targeted therapies. This study also highlights that not all R-L shunts are intrapulmonary and that different shunts will have different treatment implications. Specifically, intrapulmonary shunts are most often due to abnormal vasodilation of pulmonary vessels. Therefore, treatment focuses on: 1) reducing underlying inflammation/infection leading to pulmonary vasodilation (e.g., corticosteroids) (22); 2) vigilant PEEP titration and ventilator optimization, to prevent over dilation of pulmonary vessels, while preventing shunt from atelectasis from occurring (23, 24); and 3) avoiding pulmonary vasodilators (e.g., epoprostenol, nitric oxide, sildenafil) (25, 26). In contrast, intracardiac shunt management should lower right-sided heart pressures to prevent further shunting through an intra-atrial septum defect (e.g., PFO or ASD). Treatments include: 1) pulmonary vasodilators (e.g., inhaled nitric oxide, epoprostenol) and/or inodilators (e.g., milrinone, dobutamine) through reducing RV afterload and improving RV function (23, 27, 28); 2) lowering ventilator settings (e.g., PEEP, plateau pressures) (23, 27, 28); 3) closure or repair of an intraseptal defects (PFO, ASD) to prevent further R-L shunting (23, 27, 28); and 4) diuresis to offload RV volume overload (23, 27, 28). Regardless, diagnosing shunt in ARDS patients starts with high suspicion and prompt diagnosis.

Guidelines have promoted standardizing ARDS management, like using low-TV ventilation (29) and proning (30), among other strategies. There has also been adoption of higher PEEP in both COVID and non-COVID-ARDS. Our study is a reminder that indiscriminate use of PEEP or pulmonary vasodilators may be harmful in the wrong patient. Future work could include: 1) identifying which patients to screen for shunt; and 2) potential interventions to reduce mortality from shunts.

This study has its strengths. We confirmed that research is still feasible in the midst of a pandemic by undertaking the largest study of shunts in COVID-19 ARDS. We have designed an extensive protocol for ICU shunt workup, which was performed by intensivists during the pandemic, saving on personal protective equipment. We investigated different shunt types, cointerventions, and duration of mechanical ventilation plus other respiratory adjuncts, which is not routinely reported in ARDS literature (12). Our study reinforces the safety of intensivist and trainee TEE, given that there were no procedural complications (31), and our inter-relator scores highlight that ICU echocardiography and TCD is feasible and reliable (32, 33). We performed both unadjusted and adjusted ORs analysis using multivariable logistic regression to account for known confounders (e.g., age, illness severity) in keeping with STROBE and Newcastle-Ottawa score recommendations (21, 34).

There are several limitations to this study. The smaller size of our non-COVID arm, leading to imbalance and potential loss of statistical power; however, given the higher number of COVID-19 versus non-COVID patients, this shunt data is representative of the ICU population at the time. Patient factors such as obesity and poor windows affected our ability to perform shunt fractions, even with TEE. The presence of shunts in ARDS may be dynamic, and whereby timing of investigations (without repeat studies performed) may influence shunt frequency. Shunt frequency also may have been affected by more evidence for available treatments across subsequent waves during the pandemic. Finally, the relatively low rate of RV dysfunction and pulmonary hypertension is intriguing (although not formally measured with TAPSE or PAT) and may be because these patients underwent ultrasonographic assessments early in their course on the ventilator.

## CONCLUSIONS

There was no evidence of increased R-L shunt rates in COVID-19 compared with non-COVID and historical controls. R-L shunt presence was associated with increased in-hospital mortality for COVID-19 patients, but this did not persist for 90-day mortality or after adjusting using logistic regression.

## ACKNOWLEDGMENTS

We are grateful for the assistance and support from the University of Alberta Department of Critical Care Medicine Research Office, the University of Alberta and Royal Alexandra Hospital, Mazankowski Heart Institute, and Grey Nuns Hospital ICUs staff, nurses, and respiratory therapists and all the University of Alberta Critical Care Ultrasound Service rotators.

## Supplementary Material
