# Supplementary material for: Intrapulmonary and Intracardiac Shunts in Adult COVID-19 Versus Non-COVID Acute Respiratory Distress Syndrome ICU Patients Using Echocardiography and Contrast Bubble Studies (COVID-Shunt Study): A Prospective, Observational Cohort Study
Source: Crit Care Med. 2023 Mar 27;51(8):1023–32. doi: 10.1097/CCM.0000000000005848 (PMC10335602; doi:10.1097/CCM.0000000000005848)

**Intra-pulmonary and intra-cardiac shunts in adult COVID-19 versus non-COVID ARDS ICU patients using echocardiography and contrast bubble studies (COVID-Shunt Study): a prospective, observational cohort study**

**Supplemental Appendix**

**Table of Contents**

**Page #**

Supplemental Appendix 1: Protocol and Operational Definitions 3

Supplemental Appendix 2: STROBE Checklist 4

Supplemental Figure 1: COVID-19 Pneumonia Hypoxemia Shunt Protocol 5

Supplemental Table 1: Baseline Demographics and Clinical Characteristics 6-7

Supplemental Table 2: Outcomes and Co-Interventions 8-10

Supplemental Table 3: Ultrasonographic Findings (TTE/TCD ± TEE) 11-12

Supplemental Table 4: Kappa Inter-Rater Reliability 13

Supplemental Table 5: Multivariable Logistic Regression 14

Supplemental Figure 2: Right-to-Left Shunt Percent Positivity Rate 15

**Supplemental Appendix 1: Hypoxemia Work-up Protocol and Project Definitions**

During TTE, ECG leads were placed where possible, and we used the cardiac preset with the phased-array probe. Full echocardiography was otherwise performed, where available. An apical 4-chamber or subcostal long axis view (SubX LAX) was obtained to screen for intra-cardiac/intra-pulmonary shunt. Colour Doppler was placed over the intra-atrial septum at varying Nyquist levels to screen for an intra-atrial septum (IAS) defect from a SubX LAX view. Agitated-saline bubble studies were injected using a 3-way Luer-lock system and 10 mL saline syringes. Via either central line or peripheral intravenous we mixed and injected 0.5mL of patient’s blood, 0.5-1mL of air, and 10 mL of saline. We recorded for 30 second (representing >10 cardiac cycles) during each bubble study. We recorded at least 1-2 beats of pre-injection cardiac cycles prior to bubble injection for the purpose of counting the cardiac cycle. Bubble studies were performed at least twice (without and with simulated Valsalva).^1,2^

         If the TTE bubble study was negative, we followed with a TCD bubble study, given its increased sensitivity in detecting R-L shunts. For TCD, we used the ultrasound machine with a phased-array probe in transcranial preset. The transtemporal window was insonated with a pulse-wave spectral Doppler on the ipsilateral middle cerebral artery (MCA) at the M1 segment (3.5-5.5 mm gate) or another intra-cranial artery, if the MCA was not detectable. The same agitated saline protocol was used, as described above.^3,4^

         TEE was performed if either the TTE or TCD bubble studies were positive, or if either study was indeterminate or unattainable (technically difficult study). Full standard TEE views were recorded. Both 2D and Colour Doppler were used to interrogate the IAS for intra-cardiac shunt defects. The main views were the mid-esophageal 4-chamber, and mid-esophageal bicaval view. The agitated saline protocol was the same as described above. Colour Nyquist was adjusted from the highest to the lowest possible levels to detect the presence of intra-cardiac shunt in the IAS, either patent foramen ovale (PFO) or atrial septal defects (ASD).^1,2^

Four Canadian intensive care units participated in the study: the University of Alberta Hospital General Systems ICU, the Mazankowski Cardiovascular ICU, the Royal Alexandra Hospital ICU, and the Grey Nuns Hospital ICU.

Operational Definitions:

Myocardial infarction was defined as acute myocardial injury with clinical evidence of acute myocardial ischemia and with detection of a rise and/or fall of cardiac troponin values with at least 1 value about the 99^th^ percentile upper limit of lab testing and at least 1 of the following: symptoms of myocardial ischemia; new ischemic ECG changes; development of pathological Q waves; imaging evidence of new loss of viable myocardium or new regional wall motion abnormality in a pattern consistent with an ischemic etiology; identification of a coronary thrombus by angiography or autopsy [Thygesen Circulation 2018].^5^

Cardio-respiratory arrest was defined as the cessation of effective ventilation and circulation.^6^

Stroke was defined as central nervous system infarction of the brain, spinal cord, or retinal cell death attributable to ischemia, based on: pathological imaging; or other objective evidence of cerebral, spinal cord or retinal focal ischemic injury in a defined vascular distribution; or, clinical evidence of cerebral, spinal cord, or retinal focal ischemic injury based on symptoms on symptoms persisting >24 hours or until death, and other etiologies excluded.^7^

Systemic ischemic event was defined as a thrombosis or embolism which originate or travel through the systemic arterial circulation.^8^

Oropharyngeal/gastrointestinal bleeding/perforation was defined as either dental trauma, submucosal hematoma of pharyngeal area, jaw subluxation, oropharyngeal bleeding, gastro-esophageal perforation following TEE probe insertion.^9^

Pneumomediastinum was defined as the presence of air in the mediastinum. This condition can result from physical trauma or other situations that lead to air escaping from the lungs, airways or bowel into the chest cavity. Pneumomediastinum is a rare situation and occurs when air leaks into the mediastinum.^10^

Respiratory endotracheal tube dislodgement was defined as an ETT moving at least 2 cm, or unplanned extubation.^11^

Venous thromboembolism (VTE) was defined as: blood clots in veins, which can manifest as deep vein thrombosis (in the lower extremities) and pulmonary embolism (in the lungs).^12^

**References:**

1. Saric M, Armour AC, Arnaout MS, et al. Guidelines for the Use of Echocardiography in the Evaluation of a Cardiac Source of Embolism. *Journal of the American Society of Echocardiography*. 2016;29(1):1-42. doi:10.1016/j.echo.2015.09.011

2. Silvestry FE, Cohen MS, Armsby LB, et al. Guidelines for the Echocardiographic Assessment of Atrial Septal Defect and Patent Foramen Ovale: From the American Society of Echocardiography and Society for Cardiac Angiography and Interventions. *Journal of the American Society of Echocardiography*. 2015;28(8):910-958. doi:10.1016/j.echo.2015.05.015

3. Jauss M, Zanette E. Detection of right-to-left shunt with ultrasound contrast agent and transcranial Doppler sonography. *Cerebrovasc Dis*. 2000;10(6):490-496. doi:10.1159/000016119

4. Mojadidi MK, Roberts SC, Winoker JS, et al. Accuracy of Transcranial Doppler for the Diagnosis of Intracardiac Right-to-Left Shunt. *JACC: Cardiovascular Imaging*. 2014;7(3):236-250. doi:10.1016/j.jcmg.2013.12.011

5. Thygesen K, Alpert JS, Jaffe AS, et al. Fourth Universal Definition of Myocardial Infarction (2018). *Circulation*. 2018;138(20):e618-e651. doi:10.1161/CIR.0000000000000617

6. Patel K, Hipskind JE. Cardiac Arrest. In: *StatPearls*. StatPearls Publishing; 2022. Accessed June 7, 2022. http://www.ncbi.nlm.nih.gov/books/NBK534866/

7. Sacco RL, Kasner SE, Broderick JP, et al. An Updated Definition of Stroke for the 21st Century. *Stroke*. 2013;44(7):2064-2089. doi:10.1161/STR.0b013e318296aeca

8. Embolism – Pathologia. Accessed June 7, 2022. https://pathologia.ed.ac.uk/topic/embolism/

9. Mathur SK, Singh P. Transoesophageal Echocardiography Related Complications. *Indian J Anaesth*. 2009;53(5):567-574.

10. Kouritas VK, Papagiannopoulos K, Lazaridis G, et al. Pneumomediastinum. *J Thorac Dis*. 2015;7(Suppl 1):S44-S49. doi:10.3978/j.issn.2072-1439.2015.01.11

11. Landsperger JS, Byram JM, Lloyd BD, Rice TW, Janz DR, for the Pragmatic Critical Care Research Group. The effect of adhesive tape versus endotracheal tube fastener in critically ill adults: the endotracheal tube securement (ETTS) randomized controlled trial. *Critical Care*. 2019;23(1):161. doi:10.1186/s13054-019-2440-7

12. Venous Thromboembolism - What Is Venous Thromboembolism? | NHLBI, NIH. Accessed June 7, 2022. https://www.nhlbi.nih.gov/health/venous-thromboembolism

**Supplemental Appendix 2:** STROBE Statement—Checklist of items that should be included in reports of ***cohort studies***

|  | Item No | Recommendation | Page No. |
| --- | --- | --- | --- |
| **Title and abstract** | 1 | (*a*) Indicate the study’s design with a commonly used term in the title or the abstract | 1-3 |
|  |  | (*b*) Provide in the abstract an informative and balanced summary of what was done and what was found | 1-3 |
| Introduction | | |  |
| Background/rationale | 2 | Explain the scientific background and rationale for the investigation being reported | 4 |
| Objectives | 3 | State specific objectives, including any prespecified hypotheses | 4 |
| Methods | | |  |
| Study design | 4 | Present key elements of study design early in the paper | 4-5 |
| Setting | 5 | Describe the setting, locations, and relevant dates, including periods of recruitment, exposure, follow-up, and data collection | 4-5 |
| Participants | 6 | (*a*) Give the eligibility criteria, and the sources and methods of selection of participants. Describe methods of follow-up | 4-5 |
|  |  | (*b*) For matched studies, give matching criteria and number of exposed and unexposed | N/A |
| Variables | 7 | Clearly define all outcomes, exposures, predictors, potential confounders, and effect modifiers. Give diagnostic criteria, if applicable | 5-6 |
| Data sources/ measurement | 8* | For each variable of interest, give sources of data and details of methods of assessment (measurement). Describe comparability of assessment methods if there is more than one group | 5-6 |
| Bias | 9 | Describe any efforts to address potential sources of bias | 6-7 |
| Study size | 10 | Explain how the study size was arrived at | 7 |
| Quantitative variables | 11 | Explain how quantitative variables were handled in the analyses. If applicable, describe which groupings were chosen and why | 4-7 |
| Statistical methods | 12 | (*a*) Describe all statistical methods, including those used to control for confounding | 6-7 |
|  |  | (*b*) Describe any methods used to examine subgroups and interactions | N/A |
|  |  | (*c*) Explain how missing data were addressed | 7 |
|  |  | (*d*) If applicable, explain how loss to follow-up was addressed | 7 |
|  |  | (*e*) Describe any sensitivity analyses | 7 |
| Results | | |  |
| Participants | 13* | (a) Report numbers of individuals at each stage of study—eg numbers potentially eligible, examined for eligibility, confirmed eligible, included in the study, completing follow-up, and analysed | 7-9 |
|  |  | (b) Give reasons for non-participation at each stage | 7-9 |
|  |  | (c) Consider use of a flow diagram | 7-9 |
| Descriptive data | 14* | (a) Give characteristics of study participants (eg demographic, clinical, social) and information on exposures and potential confounders | 7-9 |
|  |  | (b) Indicate number of participants with missing data for each variable of interest | 7-9 |
|  |  | (c) Summarise follow-up time (eg, average and total amount) | 7-9 |
| Outcome data | 15* | Report numbers of outcome events or summary measures over time | 7-9 |
| Main results | 16 | (*a*) Give unadjusted estimates and, if applicable, confounder-adjusted estimates and their precision (eg, 95% confidence interval). Make clear which confounders were adjusted for and why they were included | 7-9 |
|  |  | (*b*) Report category boundaries when continuous variables were categorized | 7-9 |
|  |  | (*c*) If relevant, consider translating estimates of relative risk into absolute risk for a meaningful time period | 7-9 |
| Other analyses | 17 | Report other analyses done—eg analyses of subgroups and interactions, and sensitivity analyses | 7-9 |
| Discussion | | |  |
| Key results | 18 | Summarise key results with reference to study objectives | 10-11 |
| Limitations | 19 | Discuss limitations of the study, taking into account sources of potential bias or imprecision. Discuss both direction and magnitude of any potential bias | 10-11 |
| Interpretation | 20 | Give a cautious overall interpretation of results considering objectives, limitations, multiplicity of analyses, results from similar studies, and other relevant evidence | 10-11 |
| Generalisability | 21 | Discuss the generalisability (external validity) of the study results | 10-11 |
| Other information | | |  |
| Funding | 22 | Give the source of funding and the role of the funders for the present study and, if applicable, for the original study on which the present article is based | 11-12 |

*Give information separately for exposed and unexposed groups.

**Note:** An Explanation and Elaboration article discusses each checklist item and gives methodological background and published examples of transparent reporting. The STROBE checklist is best used in conjunction with this article (freely available on the Web sites of PLoS Medicine at http://www.plosmedicine.org/, Annals of Internal Medicine at http://www.annals.org/, and Epidemiology at http://www.epidem.com/). Information on the STROBE Initiative is available at http://www.strobe-statement.org.


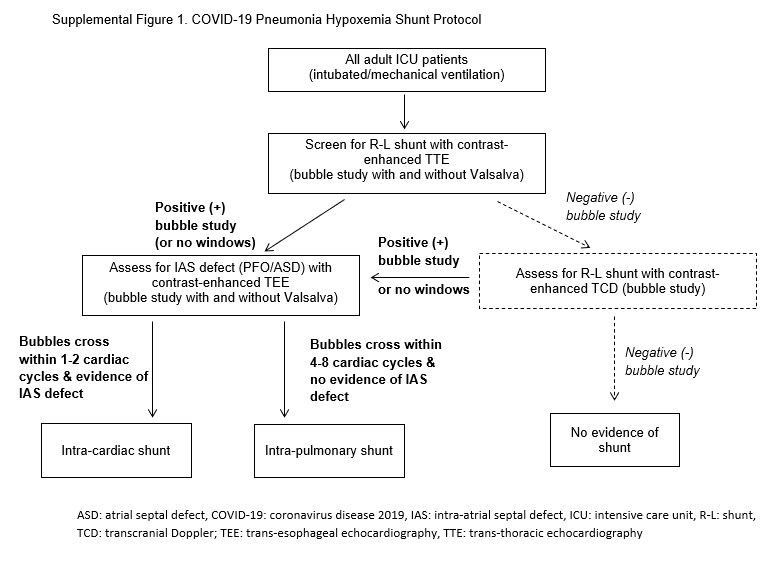


| **Supplemental Table 1**. Baseline Demographics and Clinical Characteristics | | | | | | | | |
| --- | --- | --- | --- | --- | --- | --- | --- | --- |
| Characteristic | | Non-COVID (n=44) | | | COVID-19 (n=182) | | |  |
|  | | Shunt  (N = 10) | No Shunt  (N = 34) | p-value | Shunt  (N = 31) | No Shunt  (N=151) | p-value | Inter-Cohort  p-value |
| Age (years, median [IQR]) | | 46.5 [39.0-65.0] | 56.0 [41.0-64.0] | 0.72 | 64.0 [53.0-70.0] | 58.0 [49.0-67.0] | 0.06 | 0.08 |
| Sex | Female (n, %) | 2 (20.0) | 14 (41.2) | 0.22 | 11 (35.5) | 49 (32.5) | 0.74 | 0.67 |
| Ethnicity | Caucasian (n, %) | 8 (80.0) | 24 (70.6) | 0.73 | 14 (45.2) | 83 (55.0) | 0.66 | 0.05 |
|  | Asian (n, %) | 0 (0.0) | 3 (8.8) | 0.73 | 5 (16.1) | 22 (14.6) | 0.66 | 0.05 |
|  | African (n, %) | 0 | 0 | 1.0 | 2 (6.5) | 10 (6.6) | 0.66 | 0.05 |
|  | Aboriginal (n, %) | 2 (20) | 6 (17.6) | 0.73 | 7 (22.6) | 17 (11.3) | 0.66 | 0.05 |
|  | Hispanic (n, %) | 0 | 0 | 1.0 | 2 (6.5) | 11 (7.3) | 0.66 | 0.05 |
|  | Other (n, %) | 0 | 1 (2.9%) | 0.73 | 1 (3.2) | 8 (5.3) | 0.66 | 0.05 |
| Median body mass index (kg/m^2^) | | 27.5 [18.8-40.0] | 30.1 [26.8-32.5] | 0.47 | 29.4 [26.3-31.1] | 30.7 [26.9-33.8] | 0.67 | 0.62 |
| Admitting Diagnosis | Respiratory (n, %) | 10 (100.0) | 30 (88.2) | 0.26 | 28 (90.3) | 149 (98.7) | 0.01 | 0.05 |
|  | Other (n, %): | 0 (0.0) | 12 (11.8) | <0.01 | 3 (9.7) | 2 (1.3) | 0.01 | <0.01 |
|  | Cardiovascular (n, %) | 0 (0.0) | 3 (8.8) | 0.33 | 1 (3.2) | 9 (6.0) | 0.54 | 0.74 |
|  | Gastrointestinal (n, %) | 0 (0.0) | 1 (2.9) | 0.58 | 0 (0.0) | 3 (2.0) | 0.43 | 0.78 |
|  | Neurological (n, %) | 0 (0.0) | 3 (8.8) | 0.33 | 2 (6.5) | 4 (2.6) | 0.28 | 0.28 |
|  | Trauma (n, %) | 0 (0.0) | 4 (11.8) | 0.26 | 0 (0.0) | 0 (0.0) | 1.0 | <0.001 |
|  | Genitourinary (n, %) | 0 (0.0) | 1 (2.9) | 0.58 | 0 (0.0) | 2 (1.3) | 0.52 | 0.54 |
| Charlson’s Comorbidities | Myocardial Infarction (n, %) | 0 (0.0) | 4 (11.8) | 0.26 | 4 (12.9) | 16 (10.6) | 0.71 | 0.72 |
|  | Congestive Heart Failure (n, %) | 0 (0.0) | 2 (5.9) | 0.43 | 0 (0.0) | 7 (4.6) | 0.22 | 0.83 |
|  | Peripheral Vascular Disease (n, %) | 0 (0.0) | 5 (14.7) | 0.20 | 1 (3.2) | 4 (2.6) | 0.86 | 0.01 |
|  | Strokes (n, %) | 0 (0.0) | 3 (8.8) | 0.33 | 1 (3.2) | 2 (1.3) | 0.45 | 0.06 |
|  | Dementia (n, %) | 0 (0.0) | 0 (0.0) | 1.0 | 1 (3.2) | 3 (2.0) | 0.67 | 0.32 |
|  | Chronic obstructive pulmonary disease  (n, %) | 1 (10.0) | 4 (11.8) | 0.88 | 4 (12.9) | 17 (11.3) | 0.79 | 0.97 |
|  | Connective tissue disorder (n, %) | 1 (10.0) | 4 (11.8) | 0.88 | 1 (3.2) | 0 (0.0) | 0.03 | <0.0001 |
|  | Liver disease (n, %) | 0 (0.0) | 6 (17.6) | 0.15 | 5 (16.1) | 11 (7.3) | 0.11 | 0.33 |
|  | Diabetes mellitus (n, %) | 5 (50.0) | 10 (29.4) | 0.23 | 10 (32.3) | 56 (37.1) | 0.61 | 0.79 |
|  | Hemiplegia (n, %) | 0 (0.0) | 0 (0.0) | 1.0 | 0 (0.0) | 0 (0.0) |  |  |
|  | Moderate-to-severe chronic kidney disease (n, %) | 0 (0.0) | 1 (2.9) | 0.58 | 3 (9.7) | 16 (10.6) | 0.88 | 0.09 |
|  | Solid tumor (n, %) | 1 (10.0) | 3 (8.8) | 0.91 | 1 (3.2) | 5 (3.3) | 0.98 | 0.09 |
|  | Leukemia (n, %) | 0 (0.0) | 3 (8.8) | 0.33 | 0 (0.0) | 1 (0.7) | 0.65 | 0.005 |
|  | Lymphoma (n, %) | 0 (0.0) | 1 (2.9) | 0.58 | 0 (0.0) | 1 (0.7) | 0.65 | 0.27 |
|  | Acquired immunodeficiency syndrome  (n, %) | 2 (20.0) | 0 (0.0) | 0.008 | 0 (0.0) | 0 (0.0) | 1.0 | 0.004 |
| Median Charlson’s Index (median: IQR) | | 2.0 [1.0-5.0] | 2.5 [1.0-3.0] | 0.81 | 3.0 [1.0-5.0] | 2.0 [1.0-4.0] | 0.01 | 0.98 |
| Median APACHE II (median: IQR) | | 30.5 [27.0-33.0] | 30.0 [25.0-36.0] | 0.92 | 28.0 [21.0-32.0] | 29.0 [24.0-33.0] | 0.52 | 0.10 |
| Known Pre-Existing Shunt (n, %) | | 0 (0.0) | 2 (5.9) | 0.43 | 0 (0.0) | 0 (0.0) | 1.0 | <0.01 |
| Other  Pre-Intubation  Diagnoses | Community-acquired pneumonia (n, %) | 7 (70.0) | 22 (64.7) | 0.76 | 22 (71.0) | 110 (72.8) | 0.83 | 0.38 |
|  | Hospital-acquired pneumonia (n, %) | 3 (30.0) | 15 (44.1) | 0.42 | 4 (12.9) | 12 (7.9) | 0.37 | <0.01 |
|  | Ventilator-acquired pneumonia (n, %) | 0 (0) | 5 (14.7) | 0.02 | 2 (6.5) | 9 (6.0) | 0.92 | 0.22 |
|  | Fungal pneumonia (n, %) | 2 (20.0) | 5 (14.7) | 0.69 | 1 (3.2) | 1 (0.7) | 0.21 | <0.01 |
| Blood Pressure (mmHg, median [IQR]) | | 112/64 | 111/59 | 0.57 | 128/59 | 118/59 | 0.32 | 0.84 |
| Heart Rate (bpm, median [IQR]) | | 98.0 [79.0-109.0] | 101 [87.0-109.0] | 0.99 | 70 [60.0-88.0] | 70 [60.0-88.0] | 0.94 | <0.01 |
| Oxygen Saturation (%, median [IQR]) | | 95.0 [93.0-97.0] | 95.5 [93.0-97.0] | 0.54 | 96.0 [94.0-98.0] | 96 [94.0-97.0] | 0.20 | 0.53 |
| White Blood Cell Count (x10^9^/L, median [IQR]) | | 10.3 [7.7-19.1] | 11.9 [6.5-16.4] | 0.74 | 11.7 [8.5-14.0] | 10.7 [7.8-15.2] | 0.82 | 0.98 |
| Hemoglobin (mmol/L, median [IQR]) | | 118.0 [95.0-129.0] | 95.5 [81.0-108.0] | 0.02 | 112.0 [101.0-129.0] | 116 [96.0-133.0] | 0.58 | <0.01 |
| Platelet (x10^9^/L, median [IQR]) | | 246.0 [192.0-282.0] | 177.5 [91.0-247.0] | 0.15 | 264.0 [193.0-337.0] | 223 [160.0-294.0] | 0.17 | <0.02 |
| D-Dimer (μmol/mL, median [IQR]) | | 4.0 [2.9-5.3] | 3.6 [1.9-10.0] | 0.68 | 2.1 [0.9-6.7] | 2.0 [0.9-4.5] | 0.58 | 0.06 |
| Troponin (ng/mL, median [IQR]) | | 24.0 [6.0-85.5] | 7.0 [0.1-38.0] | 0.42 | 15.0 [0.1-32.0] | 9.0 [0.2-41.0] | 0.91 | 0.93 |
| Creatinine (μmol/mL, median [IQR]) | | 85.0 [77.0-101.0] | 85.5 [60.0-124.0] | 0.99 | 74.0 [59.0-95.0] | 81.0 [64.0-108.0] | 0.24 | 0.85 |
| Pre-Intubation  Methods  Of Oxygenation | Nasal Cannula (n, %) | 9 (90.0) | 31 (91.2) | 0.91 | 25 (80.6) | 137 (90.7) | 0.10 | 0.71 |
|  | Face Mask (n, %) | 8 (80.0) | 28 (82.4) | 0.87 | 25 (80.6) | 132 (87.4) | 0.32 | 0.45 |
|  | High Flow Nasal Cannula (n, %) | 8 (80.0) | 26 (76.5) | 0.81 | 26 (83.9) | 143 (94.7) | 0.03 | <0.01 |
|  | NIPPV (n, %) | 3 (30.0) | 6 (17.6) | 0.39 | 15 (43.4) | 101 (66.9) | 0.05 | <0.01 |
| Ventilation Parameters at Time of Bubble Study | FiO2 (%, median [IQR]) | 62.5 [55.0-90.0] | 60.0 [45.0-80.0] | 0.62 | 50.0 [40.0-70.0] | 50 [45.0-75.0] | 0.36 | 0.06 |
|  | PEEP (cm H2O, median [IQR]) | 12.0 [8.0-12.0] | 12.0 [10.0-14.0] | 0.25 | 13 [11.0-16.0] | 14 [12.0-15.0] | 0.83 | <0.001 |
|  | Tidal volume (cc/kg PBW, median [IQR]) | 6.5 [5.0-9.0] | 6.8 [5.3-7.9] | 0.67 | 6.6 [5.7-8.0] | 6.1 [5.1-7.6] | 0.19 | 0.22 |
|  | Plateau Pressure (cm H2O, median [IQR]) | 29.5 [26.0-33.0] | 24.0 [21.0-28.0] | 0.01 | 26.0 [22.0-28.0] | 25.0 [22.0-28.0] | 0.90 | 0.91 |
|  | Static Compliance (cc/cm H2O, median [IQR]) | 31.0 [27.0-39.0] | 44.0 [32.0-59.0] | 0.05 | 39.0 [30.0-53.0] | 37.8 [27.0-47.0] | 0.40 | 0.39 |
|  | Deadspace (%) | 31.0 [29.0-34.0] | 27.0 [23.0-30.0] | 0.005 | 28.0 [25.0-31.0] | 28.0 [25.0-31.0] | 0.97 | 0.79 |
|  | pH (median [IQR]) | 7.3 [7.3-7.4] | 7.4 [7.3-7.5] | 0.14 | 7.4 [7.4-7.4] | 7.4 [7.3-7.4] | 0.87 | 0.96 |
|  | PaO2 (mmHg, median [IQR]) | 76.5 [63.0-93.0] | 78.0 [72.0-89.0] | 0.66 | 80.0 [71.0-93.0] | 81.0 [70.0-96.0] | 0.81 | 0.35 |
|  | PaCO2 (mmHg, median [IQR]) | 57.0 [42.0-60.0] | 44.0 [40.0-50.0] | 0.18 | 47.0 [40.0-53.0] | 46.0 [40.0-52.0] | 0.98 | 0.86 |
|  | A-a Gradient (mmHg, median [IQR]) | 300.0 [245.0-484.0] | 315.0 [173.0-408.0] | 0.92 | 229.0 [151.0-338.0] | 234.5 [168.0-358.0] | 0.37 | 0.03 |
|  | P/F Ratio (mmHg/%/100, median [IQR]) | 131.5 [80.0-167.0] | 123.0 [101.0-185.0] | 0.53 | 153.0 [136.0-200.0] | 160.0 [115.0-200.0] | 0.83 | 0.02 |

A-a: Alveolar-arterial: APACHE: Acute Physiologic Assessment and Chronic Health Evaluation; BMI: body mass index; CAP: community-acquired pneumonia; cc = cubic centimeter; cm = centimeters; COPD; chronic obstructive pulmonary disease; COVID-19: Coronavirus Disease-2019; FiO2: fraction of inspired oxygen; HAP: hospital-acquired pneumonia; H2O = water; IQR: interquartile range; kg = kilograms; m = meters; mL = milliliters; NIPPV: non-invasive positive pressure ventilation; P/F: PaO2/FiO2; PaO2: partial pressure of oxygen in arterial blood; PaCO2: partial pressure of carbon dioxide in arterial blood; PBW: predicted body weight; PEEP: positive end-expiratory pressure; pH: quantitative measure of acidity or basicity in arterial blood; SD: standard deviation; VAP: ventilator-associated pneumonia;

| **Supplemental Table 2**: Outcomes and Co-Interventions | | | | | | | | | | | | | |
| --- | --- | --- | --- | --- | --- | --- | --- | --- | --- | --- | --- | --- | --- |
| Outcome or intervention | | Non-COVID | | | | | COVID-19 | | | | | Full cohort comparison | |
|  |  | Shunt  (N=10) | Non-Shunt  (N=34) | Total  (N=44) | Mean difference: non-COVID shunt vs. non-shunt  (95% CI) | p-value | Shunt  (N=31) | Non-Shunt  (N=151) | Total  (N=182) | Mean difference:  COVID shunt vs. non-shunt  (95% CI) | p-value | Mean difference:  COVID vs. non-COVID  (95% CI) | Inter-arm  p-value |
| Mortality | In-hospital* (n, %) | 4 (40.0) | 15 (44.1) | 19 (43.2) | -4.1  [-39.0 to 30.8] | 0.82 | 17 (54.8) | 54 (35.8) | 71 (39.0) | +19.0  [+0.1 to 37.9] | 0.05 | -4.2%  [-20.3 to 11.9] | 0.30 |
|  | 90-day (n, %) | 4 (40.0) | 16 (47.1) | 20 (45.5) | -7.1  [-42.2 to 28.0] | 0.69 | 17 (54.8) | 58 (38.4) | 75 (41.4) | +16.4  [-2.6 to 35.4] | 0.10 | -4.1%  [-12.2 to 20.4] | 0.31 |
| Duration of MV (days, median: IQR) | | 7.5  [2.0-16.0] | 10.0  [5.0-17.0] | 9.0  [5.0-17.0] | -1.1  [-6.6 to 8.7] | 0.38 | 13.0  [8.0-26.0] | 15.0  [7.0-24.0] | 15.0  [8.0-25.0] | -4.6  (-13.6 to 3.5) | 0.68 | +7.5  [+0.5 to 14.5] | 0.007 |
| ICU length of stay (days, median: IQR) | | 10.5  [8.0-25.0] | 13.0  [7.0-19.0] | 12.0  [7.5-20.0] | -0.3  [-7.5 to 8.1] | 0.62 | 13.0  [10.0-27.0] | 18.0  [11.0-29.0] | 17.5  [11.0-28.0] | -6.1  (-15.3 to 3.0) | 0.26 | +8.2  [+1.0 to 15.4] | 0.007 |
| Hospital length of stay (days, median: IQR) | | 34.5  [15.0-54.0] | 20.5  [12.0-27.0] | 21.5  [13.0-32.0] | +13.1  [-2.0 to 26.0] | 0.13 | 20.0  [13.0-33.0] | 26.0  [16.0-42.0] | 25.5  [16.0-42.0] | -8.1  (-19.4 to 3.3) | 0.09 | +7.2  [-1.9 to 16.3] | 0.11 |
| In-hospital  complication | Myocardial Infarction (n, %) | 0 (0.0) | 1 (2.9) | 1 (2.3) | -2.9  [-13.0 to 8.0] | 0.58 | 0 (0.0) | 7 (4.6) | 7 (3.8) | -4.6  [-12.0 to 2.8] | 0.11 | +1.5  [-4.6 to 7.6] | 0.31 |
|  | Stroke (n, %) | 0 (0.0) | 1 (2.9) | 1 (2.3) | -2.9  [-13.0 to 8.0] | 0.58 | 3 (9.7) | 3 (2.0) | 6 (3.3) | +7.7  [1.0 to 14.6] | 0.01 | +1.0  [-4.7 to 6.7] | 0.37 |
|  | Other ischemia (n, %) | 0 (0.0) | 0 (0.0) | 0 (0.0) | 0.0  [0.0 to 0.0] | 1.0 | 0 (0.0) | 3 (2.0) | 3 (1.6) | -2.0  [-6.9 to 2.9] | 0.21 | +1.6  [-2.2 to 5.2] | 0.22 |
|  | Oropharyngeal Bleed (n, %) | 0 (0.0) | 0 (0.0) | 0 (0.0) | 0.0  [0.0 to 0.0] | 1.0 | 0 (0.0)** | 0 (0.0)** | 0 (0.0) | 0.0  [0.0 to 0.0] | 1.0 | 0.0  [0.0 to 0.0] | 1.0 |
|  | GI bleed (n, %) | 0 (0.0) | 2 (5.9) | 2 (4.5) | -5.9  [-21.0 to 9.0] | 0.43 | 3 (9.7)** | 10 (6.6)** | 13 (7.1) | +3.1  [-6.8 to 13.0] | 0.27 | +2.6  [-5.6 to 10.8] | 0.53 |
|  | GI perforation (n, %) | 0 (0.0) | 0 (0.0) | 0 (0.0) | 0.0  [0.0 to 0.0] | 1.0 | 0 (0.0)** | 1 (0.7)** | 1 (0.5) | -0.7  [-3.6 to 2.3] | 0.32 | +0.5  [-0.2 to 2.0] | 0.62 |
|  | Pneumo-mediastinum (n, %) | 0 (0.0) | 0 (0.0) | 0 (0.0) | 0.0  [0.0 to 0.0] | 1.0 | 2 (6.5)** | 7 (4.6)** | 9 (4.9) | +1.9  [-6.5 to 10.3] | 0.33 | +4.9  [-0.2 to 8.1] | 0.13 |
|  | Airway dislodgement (n, %) | 0 (0.0) | 0 (0.0) | 0 (0.0) | 0.0  [0.0 to 0.0] | 1.0 | 0 (0.0) | 0 (0.0) | 0 (0.0) | 0.0  [0.0 to 0.0] | 1.0 | 0.0  [0.0 to 0.0] | 1.0 |
|  | DVT (n, %) | 0 (0.0) | 0 (0.0) | 0 (0.0) | 0.0  [0.0 to 0.0] | 1.0 | 0 (0.0) | 7 (4.6) | 7 (3.8) | -4.6  [-12.0 to 2.8] | 0.11 | +3.8  [-1.0 to 6.6] | 0.17 |
|  | PE (n, %) | 0 (0.0) | 1 (2.9) | 1 (2.3) | -2.9  [-13.0 to 8.0] | 0.58 | 9 (29.0) | 16 (10.6) | 25 (13.7) | +18.4  [5.1 to 31.7] | 0.003 | +11.4  [+1.0 to 21.9] | 0.03 |
| ARDS treatment | Proning (n, %) | 5 (50.0) | 14 (41.2) | 19 (43.2) | +8.8  [-4.4 to 26.0] | 0.31 | 27 (87.1) | 114 (75.5) | 141 (77.5) | +11.6  [-4.5 to 27.7] | 0.08 | +32.3  [+17.1 to 47.5] | <0.01 |
|  | APRV (n, %) | 1 (10.0) | 3 (8.8) | 4 (9.1) | +1.2  [-19.5 to 21.5] | 0.45 | 4 (12.9) | 21 (13.9) | 25 (13.7) | -10.0  [-14.3 to 12.3] | 0.44 | +4.6  [-6.4 to 15.6] | 0.41 |
|  | Inhaled Vasodilator (n, %) | 3 (30.0) | 5 (14.7) | 8 (18.2) | +15.3  [-11.9 to 42.3] | 0.14 | 6 (19.4) | 31 (20.5) | 37 (20.3) | -1.1  [-16.7 to 14.5] | 0.88 | +2.1  [-11.0 to 15.2] | 0.75 |
|  | ECLS/ECMO (n, %) | 0 (0.0) | 3 (8.8) | 3 (6.8) | -8.8  [-26.5 to 8.6] | 0.17 | 4 (12.9) | 25 (16.6) | 29 (15.9) | -3.7  [-17.9 to 10.5] | 0.61 | +9.1  [-2.4 to 20.6] | 0.06 |
| Sedation & analgesia | Propofol (n, %) | 9 (90.0) | 33 (97.1) | 42 (95.5) | -7.1  [-21.7 to 7.5] | 0.34 | 31 (100.0) | 148 (98.0) | 179 (98.4) | +2.0  [-2.9 to 6.9] | 0.21 | +2.9  [-3.5 to 9.3] | 0.24 |
|  | Dexmedetomidine (n,%) | 0 (0.0) | 6 (17.6) | 6 (13.6) | -17.6  [-41.8 to 6.6] | 0.15 | 7 (22.6) | 45 (29.8) | 52 (28.6) | -7.2  [-24.7 to 10.3] | 0.21 | +15.0  [+1.6 to 30.8] | 0.04 |
|  | Hydromorphone (n, %) | 10 (100.0) | 34 (100.0) | 44 (100.0) | 0.0  [0.0 to 0.0] | 1.0 | 31 (100.0) | 150 (99.3) | 181 (99.5) | -0.7  [-3.6 to 2.2] | 0.32 | -0.7  [-3.2 to 1.8] | 0.62 |
|  | Midazolam (n, %) | 6 (60.0) | 16 (47.1) | 22 (50.0) | +12.9  [-22.4 to 48.2] | 0.24 | 19 (61.3) | 113 (74.8) | 132 (72.5) | +13.5  [-0.4 to 30.8] | 0.06 | +22.5  [+7.2 to 37.8] | 0.002 |
|  | Rocuronium (n, %) | 4 (40.0) | 13 (38.2) | 17 (38.6) | +1.8  [-36.1 to 32.5] | 0.46 | 24 (77.4) | 104 (68.9) | 128 (70.3) | +8.5  [-9.2 to 26.2] | 0.17 | +31.7  [+15.9 to 37.5] | <0.01 |
|  | Cisatracurium (n, %) | 1 (10.0) | 1 (2.9) | 2 (4.5) | +7.1  [-7.5 to 21.7] | 0.17 | 4 (12.9) | 18 (11.9) | 22 (12.1) | +1.0  [-13.6 to 11.6] | 0.44 | +7.6  [-2.5 to 17.7] | 0.15 |
| Renal replacement | IHD (n, %) | 0 (0.0) | 1 (2.9) | 1 (2.3) | -2.9  [-13.0 to 8.0] | 0.58 | 1 (3.2) | 6 (4.0) | 7 (3.8) | -0.8  [-8.3 to 6.7] | 0.42 | +1.5  [-4.6 to 7.6] | 0.61 |
|  | CRRT (n, %) | 1 (10.0) | 3 (8.8) | 4 (9.1) | -1.2  [-21.5 to 19.1] | 0.45 | 1 (3.2) | 13 (8.6) | 14 (7.7) | -5.4  [-15.7 to 4.9] | 0.15 | -1.4  [-10.3 to 7.5] | 0.76 |
| Shock subtype | Distributive (n, %) | 8 (80.0) | 31 (91.2) | 39 (88.6) | -11.2  [-33.6 to 11.2] | 0.16 | 30 (96.8) | 135 (89.4) | 165 (90.7) | +7.4  [-3.9 to 18.7] | 0.10 | +2.1  [-7.8 to 11.9] | 0.68 |
|  | Hypovolemic (n, %) | 2 (20.0) | 3 (8.8) | 5 (11.4) | +11.2  [-11.6 to 33.6] | 0.16 | 2 (6.5) | 13 (8.6) | 15 (8.2) | -2.1  [-12.7 to 8.5] | 0.35 | -3.2  [-12.5 to 6.1] | 0.69 |
|  | Obstructive (n, %) | 0 (0.0) | 3 (8.8) | 3 (6.8) | -8.8  [-26.6 to 9.0] | 0.33 | 4 (12.9) | 3 (2.0) | 7 (3.8) | +10.9  [+3.5 to 18.3] | 0.002 | +3.0  [-3.7 to 9.7] | 0.39 |
|  | Cardiogenic (n, %) | 4 (40.0) | 4 (11.8) | 8 (18.2) | +28.2  [1.0 to 55.0] | 0.04 | 4 (12.9) | 14 (9.3) | 18 (9.9) | +3.6  [-15.2 to 8.0] | 0.27 | -8.3  [-18.8 to 2.2] | 0.12 |
|  | None (n, %) | 0 (0.0) | 0 (0.0) | 0 (0.0) | 0.0  [0.0 to 0.0] | 1.0 | 0 (0.0) | 0 (0.0) | 0 (0.0) | 0.0  [0.0 to 0.0] | 1.0 | 0.0  [0.0 to 0.0] | 1.0 |
| Vasoactives  use | No vasoactives (n, %) | 1 (10.0) | 1 (2.9) | 2 (4.5) | +7.1  [-7.5 to 21.7] | 0.17 | 1 (3.2) | 14 (9.3) | 15 (8.2) | -6.1  [-16.7 to 4.5] | 0.26 | +3.7  [-5.0 to 12.4] | 0.40 |
|  | Norepinephrine (n, %) | 9 (90.0) | 33 (97.1) | 42 (95.5) | -7.1  [-21.7 to 7.5] | 0.35 | 29 (93.5) | 137 (90.7) | 166 (91.2) | +2.8  [-8.1 to 13.8] | 0.31 | -4.3  [-13.2 to 4.6] | 0.35 |
|  | Epinephrine (n, %) | 1 (10.0) | 4 (11.8) | 5 (11.4) | -1.8  [-24.2 to 20.6] | 0.44 | 5 (16.1) | 8 (5.3) | 13 (7.1) | +10.8  [+1.0 to 20.8] | 0.02 | -4.3  [-13.2 to 4.6] | 0.35 |
|  | Dobutamine (n, %) | 3 (30.0) | 3 (8.8) | 6 (13.6) | +21.2  [3.0 to 45.4] | 0.04 | 1 (3.2) | 9 (6.0) | 10 (5.5) | -2.8  [-11.6 to 6.0] | 0.27 | -8.1  [-0.1 to 16.5] | 0.06 |
|  | Dopamine (n, %) | 0 (0.0) | 0 (0.0) | 0 (0.0) | 0.0  [0.0 to 0.0] | 1.0 | 0 (0.0) | 0 (0.0) | 0 (0.0) | 0.0  [0.0 to 0.0] | 1.0 | 0.0  [0.0 to 0.0] | 1.0 |
|  | Phenylephrine (n, %) | 1 (10.0) | 1 (2.9) | 2 (4.5) | +7.1  [-8.0 to 22.0] | 0.17 | 3 (9.7) | 4 (2.6) | 7 (3.8) | +7.1  [-0.3 to 14.5] | 0.06 | -0.7  [-7.1 to 5.7] | 0.83 |
|  | Vasopressin (n, %) | 4 (40.0) | 21 (61.8) | 25 (56.8) | -21.8  [-56.7 to 13.1) | 0.11 | 15 (48.4) | 63 (41.7) | 78 (42.9) | +6.7  [-12.4 to 25.8] | 0.25 | -13.9  [-2.5 to 30.3] | 0.10 |
|  | Milrinone (n, %) | 1 (10.0) | 0 (0.0) | 1 (2.3) | +10.0  [0.5 to 25.0] | 0.03 | 1 (3.2) | 3 (2.0) | 4 (2.2) | +1.2  [-4.5 to 6.9] | 0.34 | -0.1  [-5.0 to 4.8] | 0.98 |
| Steroid use | None (n, %) | 1 (10.0) | 12 (35.3) | 13 (29.5) | -25.3  [-57.5 to 6.9] | 0.12 | 0 (0.0) | 1 (0.7). | 1 (0.5) | -0.7  [-2.0 to 0.1] | 0.65 | -29.0  [-43.0 to -15.0] | <0.0001 |
|  | Dexamethasone (n, %) | 6 (60.0) | 6 (17.6) | 12 (27.3) | +42.4  [11.0 to 73.8] | 0.008 | 31 (100.0) | 150 (99.3) | 181 (99.5) | 0.7  [-0.1 to 2.0] | 0.65 | +72.2  [56.0 to 88.4] | <0.0001 |
|  | Hydrocortisone (n, %) | 2 (20.0) | 12 (35.3) | 14 (31.8) | -15.3  [-48.1 to 17.5] | 0.36 | 2 (6.5) | 7 (4.6) | 9 (4.9) | -1.9  [-11.2 to 7.4] | 0.67 | -26.9  [-41.4 to -12.4] | <0.0001 |
|  | Prednisone (n, %) | 2 (20.0) | 5 (14.7) | 7 (15.9) | +5.3  [-20.5 to 31.1] | 0.69 | 1 (3.2) | 8 (5.3) | 9 (4.9) | -2.1  [-9.3 to 5.1] | 0.63 | -0.11  [-22.3 to -0.3] | 0.01 |
|  | Methylprednisone (n, %) | 1 (10.0) | 5 (14.7) | 6 (13.6) | -4.7  [-28.9 to 19.5] | 0.70 | 0 (0.0) | 6 (4.0) | 6 (3.3) | -4.0  [-10.9 to 2.9] | 0.26 | -10.3  [-20.8 to -0.2] | 0.006 |

APRV: airway-pressure release ventilation; COVID-19: Coronavirus Disease-2019; CI: confidence interval; CRRT: continuous renal replacement therapy; DVT: deep vein thrombosis; ECLS: extracorporeal life support; ECMO: extracorporeal membrane oxygenation; GI: gastrointestinal; ICU: intensive care unit; IHD: intermittent hemodialysis; IQR: interquartile range; MV = mechanical ventilation; n = number; PE = pulmonary embolism; SD: standard deviation;

*Primary outcome

**TEE not performed on any patient with pre-existing oropharyngeal/GI bleeding or pneumomediastinum; there were no post-TEE procedural complications from oropharyngeal/GI bleeding or pneumomediastinum

| **Supplemental Table 3.** Ultrasonographic Findings (TTE/TCD ± TEE) | | | | | |
| --- | --- | --- | --- | --- | --- |
| Finding | | Non-COVID | COVID-19 | Risk Difference:  COVID vs. non-COVID (95% CI) | p-value |
| TTE Bubble Study | Positive (n, %) | 5/44 (11.4) | 20/178 (11.2) | -1.3 [-13.3 to 8.3] | 0.98 |
|  | Negative (with and without Valsalva) | 30/44 (68.2) | 136/178 (76.4) | +8.2 [-6.1 to 22.5] | 0.26 |
|  | Unable to obtain (n, %) | 9/44 (20.5) | 22/178 (12.4) | -8.1 [-19.6 to 3.4] | 0.17 |
| TCD Bubble Study | Positive (n, %) | 1/30 (3.3) | 4/142 (2.8) | -0.5 [-7.1 to 6.1] | 0.88 |
|  | Negative (with and without Valsalva) | 20/30 (66.7) | 128/142 (90.1) | +23.4 [9.7 to 37.1] | 0.0007 |
|  | Unable to obtain (n, %) | 9/30 (30.0) | 10/142 (7.0) | -23.0 [-10.7 to 35.3] | 0.0003 |
| TEE Bubble Study | Positive (n, %) | 6/11 (54.5) | 23/58 (39.7) | -14.8 [-46.6 to 17.0] | 0.36 |
|  | Negative (with and without Valsalva) | 5/11 (45.5) | 35/58 (60.3) | +14.8 [-17.0 to 46.6] | 0.36 |
| Total R-L Shunts (n, %) | | 10/44 (22.7) | 31/182 (17.0) | -5.7 [-18.4 to 7.0] | 0.38 |
| Shunt Etiology | Intra-Cardiac (IAS defect, n, %) | 7/10 (70.0) | 12/31 (38.7) | -31.3 [-66.8 to 4.2] | 0.08 |
|  | PFO (n, %) | 7/10 (70.0) | 12/31 (38.7) | -31.3 [-66.8 to 4.2] | 0.08 |
|  | ASD (n, %) | 0/10 (0.0) | 0/31 (0.0) | 0.0 [0.0 to 0.0] | 1.0 |
|  | Intra-Pulmonary (n, %) | 3/10 (30.0) | 19/31 (61.2) | +31.2 [-4.4 to 66.8] | 0.08 |
| LV Function | Severely depressed (LVEF: <30%, n, %) | 4/44 (9.1) | 4/182 (2.2) | -6.9 [-13.0 to 8.1] | 0.73 |
|  | Depressed (LVEF: 30-50%, n, %) | 3/44 (6.8) | 17/182 (9.4) | +2.6 [-6.8 to 12.0] | 0.60 |
|  | Normal function (LVEF: 50-70%, n, %) | 35/44 (79.5) | 153/182 (89.0) | +9.5 [-1.5 to 20.5] | 0.47 |
|  | Hyperdynamic (LVEF: >70%, n, %) | 2/44 (4.5) | 8/182 (4.4) | -0.1 [-6.9 to 6.7] | 0.97 |
| RV Function | Severe dysfunction (n, %) | 2/44 (4.5) | 2/182 (1.0) | -3.5 [-7.7 to 1.0] | 0.12 |
|  | Moderate dysfunction (n, %) | 2/44 (4.5) | 6/182 (3.3) | -1.2 [-7.3 to 4.9] | 0.69 |
|  | Mild dysfunction (n, %) | 5/44 (11.4) | 18/182 (9.9) | -1.5 [-11.5 to 8.5] | 0.77 |
|  | Normal function (n, %) | 35/44 (79.5) | 155/182 (85.2) | +5.7 [-6.3 to 17.7] | 0.36 |
|  | Hyperdynamic (n, %) | 0/44 (0.0) | 1/182 (0.5) | 0.0 [0.0 to 0.0] | 1.0 |
| RV Size | Normal (n, %) | 36/44 (81.8) | 152/182 (83.5) | +1.7 [-10.6 to 14.0] | 0.79 |
|  | Mild dilation (RV < 2/3 LV size, n, %) | 1/44 (2.3) | 11/182 (6.0) | +3.7 [-3.7 to 11.1] | 0.32 |
|  | Moderate dilation (RV:LV size 1:1, n, %) | 6/44 (13.6) | 15/182 (8.2) | -5.4 [-14.9 to 4.1] | 0.27 |
|  | Severe dilation (RV > LV size, n, %) | 1/44 (2.3) | 4/182 (2.2) | -0.1 [-5.0 to 4.8] | 0.98 |
|  | Septal flattening (n, %) | 1/44 (2.3) | 2/182 (1.0) | -1.3 [-5.0 to 2.4] | 0.54 |
| Pericardial Effusion | No Effusion (n, %) | 36/44 (81.8) | 164/182 (90.1) | +8.3 [-2.2 to 18.8] | 0.12 |
|  | Trace/Small Effusion [0-1 cm] (n, %) | 7/44 (15.9) | 17/182 (9.3) | -6.6 [-16.7 to 3.5] | 0.20 |
|  | Moderate Effusion [1-2 cm] (n, %) | 1/44 (2.3) | 0/182 (0.0) | -2.3 [-4.5 to 0.1] | 0.19 |
|  | Large Effusion [>2 cm] n, %) | 0/44 (0.0) | 1/182 (0.5) | +0.5 [-1.2 to 2.6] | 1.0 |
|  | Pericardial tamponade | 0/44 (0.0) | 1/182 (0.5) | +0.5 [-1.2 to 2.6] | 1.0 |
| Aortic valve | No aortic stenosis | 44/44 (100.0) | 179/182 (98.4) | -1.6 [-5.3 to 2.1] | 1.0 |
|  | Mild aortic stenosis | 0/44 (0.0) | 0/182 (0.0) | 0.0 [0.0 to 0.0] | 1.0 |
|  | Moderate aortic stenosis | 0/44 (0.0) | 2/182 (1.0) | +1.0 [-1.9 to 3.9] | 1.0 |
|  | Severe aortic stenosis | 0/44 (0.0) | 1/182 (0.5) | +0.5 [-1.2 to 2.6] | 1.0 |
|  | No aortic regurgitation | 42/44 (95.5) | 154/182 (83.5) | -12.0 [-0.5 to 23.5] | 0.06 |
|  | Trace-mild aortic regurgitation | 2/44 (4.5) | 26/182 (14.3) | +9.8 [-1.1 to 20.7] | 0.08 |
|  | Moderate aortic regurgitation | 0/44 (0.0) | 2/182 (1.0) | +1.0 [-1.9 to 3.9] | 1.0 |
|  | Severe aortic regurgitation | 0/44 (0.0) | 0/182 (0.0) | 0.0 [0.0 to 0.0] | 1.0 |
| Mitral valve | No mitral stenosis | 43/44 (97.7) | 179/182 (98.4) | +0.7 [-3.6 to 5.0] | 0.78 |
|  | Mild mitral stenosis | 1/44 (2.3) | 3/182 (1.6) | -0.7 [-5.0 to 3.6] | 0.78 |
|  | Moderate mitral stenosis | 0/44 (0.0) | 0/182 (0.0) | 0.0 [0.0 to 0.0] | 1.0 |
|  | Severe mitral stenosis | 0/44 (0.0) | 0/182 (0.0) | 0.0 [0.0 to 0.0] | 1.0 |
|  | No mitral regurgitation | 32/44 (72.7) | 137/182 (75.3) | +2.6 [-11.7 to 16.9] | 0.53 |
|  | Trace-mild mitral regurgitation | 12/44 (27.3) | 34/182 (18.7) | -8.6 [-21.9 to 4.7] | 0.20 |
|  | Moderate mitral regurgitation | 0/44 (0.0) | 10/182 (5.5) | +5.5 [-1.3 to 12.3] | 0.22 |
|  | Severe mitral regurgitation | 0/44 (0.0) | 1/182 (0.5) | +0.5 [-1.2 to 2.6] | 1.0 |
| Tricuspid valve | No tricuspid stenosis | 44/44 (100.0) | 182/182 (100.0) | 0.0 [0.0 to 0.0] | 1.0 |
|  | Mild tricuspid stenosis | 0/44 (0.0) | 0/182 (0.0) | 0.0 [0.0 to 0.0] | 1.0 |
|  | Moderate tricuspid stenosis | 0/44 (0.0) | 0/182 (0.0) | 0.0 [0.0 to 0.0] | 1.0 |
|  | Severe tricuspid stenosis | 0/44 (0.0) | 0/182 (0.0) | 0.0 [0.0 to 0.0] | 1.0 |
|  | No tricuspid regurgitation | 20/44 (45.5) | 85/182 (46.7) | +1.2 [-15.2 to 17.6] | 0.88 |
|  | Trace-mild tricuspid regurgitation | 19/44 (43.2) | 86/182 (47.2) | +4.0 [-12.4 to 20.4] | 0.63 |
|  | Moderate tricuspid regurgitation | 5/44 (11.4) | 11/182 (6.0) | -5.4 [-13.8 to 3.0] | 0.22 |
|  | Severe tricuspid regurgitation | 0/44 (0.0) | 0/182 (0.0) | 0.0 [0.0 to 0.0] | 1.0 |
| Pulmonic valve | No pulmonic stenosis | 44/44 (100.0) | 182/182 (100.0) | 0.0 [0.0 to 0.0] | 1.0 |
|  | Mild pulmonic stenosis | 0/44 (0.0) | 0/182 (0.0) | 0.0 [0.0 to 0.0] | 1.0 |
|  | Moderate pulmonic stenosis | 0/44 (0.0) | 0/182 (0.0) | 0.0 [0.0 to 0.0] | 1.0 |
|  | Severe pulmonic stenosis | 0/44 (0.0) | 0/182 (0.0) | 0.0 [0.0 to 0.0] | 1.0 |
|  | No pulmonic regurgitation | 42/44 (95.5) | 171/182 (94.0) | -1.5 [-9.1 to 6.1] | 0.70 |
|  | Trace-mild pulmonic regurgitation | 2/44 (4.5) | 11/182 (6.0) | +1.5 [-6.1 to 9.1] | 0.70 |
|  | Moderate pulmonic regurgitation | 0/44 (0.0) | 0/182 (0.0) | 0.0 [0.0 to 0.0] | 1.0 |
|  | Severe pulmonic regurgitation | 0/44 (0.0) | 0/182 (0.0) | 0.0 [0.0 to 0.0] | 1.0 |
| Pulmonary hypertension | No pulmonary hypertension* | 35/44 (79.5) | 154/182 (84.6) | +5.1 [-7.1 to 17.3] | 0.41 |
|  | Mild pulmonary hypertension | 4/44 (9.1) | 15/182 (8.2) | -0.9 [-10.0 to 8.2] | 0.86 |
|  | Moderate pulmonary hypertension | 4/44 (9.1) | 12/182 (6.6) | -2.5 [-11.0 to 6.0] | 0.56 |
|  | Severe pulmonary hypertension | 1/44 (2.3) | 1/182 (0.5) | -1.8 [-4.8 to 1.2] | 0.27 |

ASD: atrial septal defect; CI: confidence interval; cm = centimeters; IAS: intra-atrial septal LV: left ventricle; LVEF: left ventricular ejection fraction; PFO: patent foramen ovale; RV: right ventricle; TEE: transesophageal echocardiography; TTE: transthoracic echocardiography;

* There may have been insufficient TR to quantify degree of pulmonary hypertension

**Supplemental Table 4:** Inter-rater reliability (kappa) of right-to-left shunt and intra-pulmonary vs. intra-cardiac shunts using TTE/TCD/TEE and contrast bubble studies (all studies)

| Positive bubble study (TTE/TEE) |  | | |
| --- | --- | --- | --- |
| Inter-relater reliability | Rater #2 | | |
| Rater #1 | Positive bubble study | Negative bubble study | Total |
| Positive bubble study | 35 | 2 | 37 |
| Negative bubble study | 0 | 189 | 189 |
| Total | 35 | 191 | 226 |
|  |  |  |  |
| Rater agreement (observed) | 35 | 189 | 224 |
| Rater agreement by chance (expected) | 5.73 | 159.06 | 163.79 |
|  |  |  |  |
|  | Kappa | 95% CI (lower limit) | 95% CI (upper limit) |
| Cohen’s kappa statistic | 0.968 | 0.923 | 1.00 |
|  |  |  |  |
| Positive bubble study (TCD) |  |  |  |
| Inter-relater reliability | Rater #2 | | |
| Rater #1 | Positive bubble study | Negative bubble study | Total |
| Positive bubble study | 2 | 0 | 2 |
| Negative bubble study | 0 | 189 | 189 |
| Total | 2 | 189 | 191 |
|  |  |  |  |
| Rater agreement (observed) | 2 | 189 | 191 |
| Rater agreement by chance (expected) | 0.021 | 187.02 | 187.04 |
|  |  |  |  |
|  | Kappa | 95% CI (lower limit) | 95% CI (upper limit) |
| Cohen’s kappa statistic | 1.00 | 1.00 | 1.00 |
|  |  |  |  |
|  |  |  |  |
| PFO or ASD identified (TTE/TEE) | Rater #2 |  |  |
| Rater #1 | PFO/ASD present | PFO/ASD absent | Total |
| PFO/ASD present | 21 | 1 | 22 |
| PFO/ASD absent | 5 | 193 | 198 |
| Total | 26 | 194 | 220 |
|  |  |  |  |
| Rater agreement (observed) | 21 | 193 | 214 |
| Rater agreement by chance (expected) | 2.10 | 173.1 | 175.8 |
|  |  |  |  |
|  | Kappa | 95% CI (lower limit) | 95% CI (upper limit) |
| Cohen’s kappa statistic | 0.864 | 0.749 | 0.970 |

ASD: atrial septal defect; CI = confidence interval; PFO: patent foramen ovale; TCD: transcranial Doppler; TEE: transesophageal echocardiography; TTE: transthoracic echocardiography;

**Supplemental Table 5:** Multivariable logistic regression (unadjusted and adjusted odds ratios)

|  | **90-Day Mortality** | | |
| --- | --- | --- | --- |
| **Risk Factor** | **Prevalence (n=226, %)** | **Unadjusted Odds Ratio (95% CI)** | **Adjusted Odds Ratio (95% CI)** |
| Intubated ARDS (any shunt) | 41 (18.1) | 1.56 (0.79-3.08) | 1.22 (0.57-2.57)** |
| - Adjusted for age |  |  | 1.01 (0.99-1.04) |
| - Adjusted for sex |  |  | 1.17 (0.62-2.18) |
| - Adjusted for APACHE |  |  | 1.02 (0.98-1.06) |
| - Adjusted for Charlson’s |  |  | 1.28 (1.09-1.52) |
| Intubated ARDS (intra-cardiac shunt) | 19 (8.4) | 1.58 (0.62-4.06) | 1.78 (0.64-4.94)** |
| - Adjusted for age |  |  | 1.02 (0.99-1.04) |
| - Adjusted for sex |  |  | 1.18 (0.63-2.22) |
| - Adjusted for APACHE |  |  | 1.01 (0.98-1.05) |
| - Adjusted for Charlson’s |  |  | 1.29 (1.09-1.52) |
| Intubated ARDS (intra-pulmonary shunt) | 22 (9.7) | 1.42 (0.59-3.42) | 0.82 (0.30-2.24)** |
| - Adjusted for age |  |  | 1.02 (0.99-1.04) |
| - Adjusted for sex |  |  | 1.17 (0.62-2.18) |
| - Adjusted for APACHE |  |  | 1.01 (0.98-1.06) |
| - Adjusted for Charlson’s |  |  | 1.29 (1.09-1.52) |
| **Risk Factor** | **Prevalence (n=182, %)** | **Unadjusted Odds Ratio (95% CI)** | **Adjusted Odds Ratio (95% CI)** |
| COVID-19 ARDS (any shunt) | 31 (17.0) | 1.96 (0.88-4.20) | 1.53 (0.64-3.67)** |
| - Adjusted for age |  |  | 1.02 (0.99-1.06) |
| - Adjusted for sex |  |  | 0.77 (0.37-1.60) |
| - Adjusted for APACHE |  |  | 1.06 (1.01-1.11) |
| - Adjusted for Charlson’s |  |  | 1.15 (0.95-1.40) |
| COVID-19 ARDS (intra-cardiac shunt) | 12 (6.6) | 3.04 (0.88-10.51) | 2.83 (0.75-10.70)** |
| - Adjusted for age |  |  | 1.03 (0.99-1.06) |
| - Adjusted for sex |  |  | 0.81 (0.39-1.67) |
| - Adjusted for APACHE |  |  | 1.05 (1.00-1.10) |
| - Adjusted for Charlson’s |  |  | 1.16 (0.96-1.41) |
| COVID-19 ARDS (intra-pulmonary shunt) | 19 (10.4) | 1.31 (0.59-3.42) | 0.93 (0.31-2.80)** |
| - Adjusted for age |  |  | 1.03 (0.99-1.06) |
| - Adjusted for sex |  |  | 0.79 (0.38-1.63) |
| - Adjusted for APACHE |  |  | 1.05 (1.01-1.10) |
| - Adjusted for Charlson’s |  |  | 1.16 (0.95-1.40) |
| **Risk Factor** | **Prevalence (n=44, %)** | **Unadjusted Odds Ratio (95% CI)** | **Adjusted Odds Ratio (95% CI)** |
| Non-COVID ARDS (any shunt) | 10 (22.7) | 0.75 (0.18-3.14) | 0.75 (0.08-6.79)** |
| - Adjusted for age |  |  | 0.98 (0.89-1.08) |
| - Adjusted for sex |  |  | 3.65 (0.67-20.04) |
| - Adjusted for APACHE |  |  | 0.85 (0.74-0.97) |
| - Adjusted for Charlson’s |  |  | 2.64 (1.12-6.62) |
| Non-COVID ARDS (intra-cardiac shunt) | 7 (15.9) | 0.42 (0.07-2.46) | 0.37 (0.03, 5.37)** |
| - Adjusted for age |  |  | 0.98 (0.89-1.07) |
| - Adjusted for sex |  |  | 3.87 (0.70-21.47) |
| - Adjusted for APACHE |  |  | 0.85 (0.74-0.97) |
| - Adjusted for Charlson’s |  |  | 2.63 (1.17-5.91) |
| Non-COVID ARDS (intra-pulmonary shunt) | 3 (6.8) | 2.56 (0.21-30.57) | 9.96 (0.07-1419.34)** |
| - Adjusted for age |  |  | 0.95 (0.85-1.07) |
| - Adjusted for sex |  |  | 5.04 (0.79-32.05) |
| - Adjusted for APACHE |  |  | 0.85 (0.75-0.97) |
| - Adjusted for Charlson’s |  |  | 3.08 (1.19-7.98) |

**adjusted for: patient’s age, sex, APACHE II, Charlson’s Cormorbidity Index (combined)

APACHE: Acute Physiologic Assessment and Chronic Health Evaluation; ARDS: acute respiratory distress syndrome; CI = confidence interval; COVID-2019: Coronavirus Disease-2019; n = number


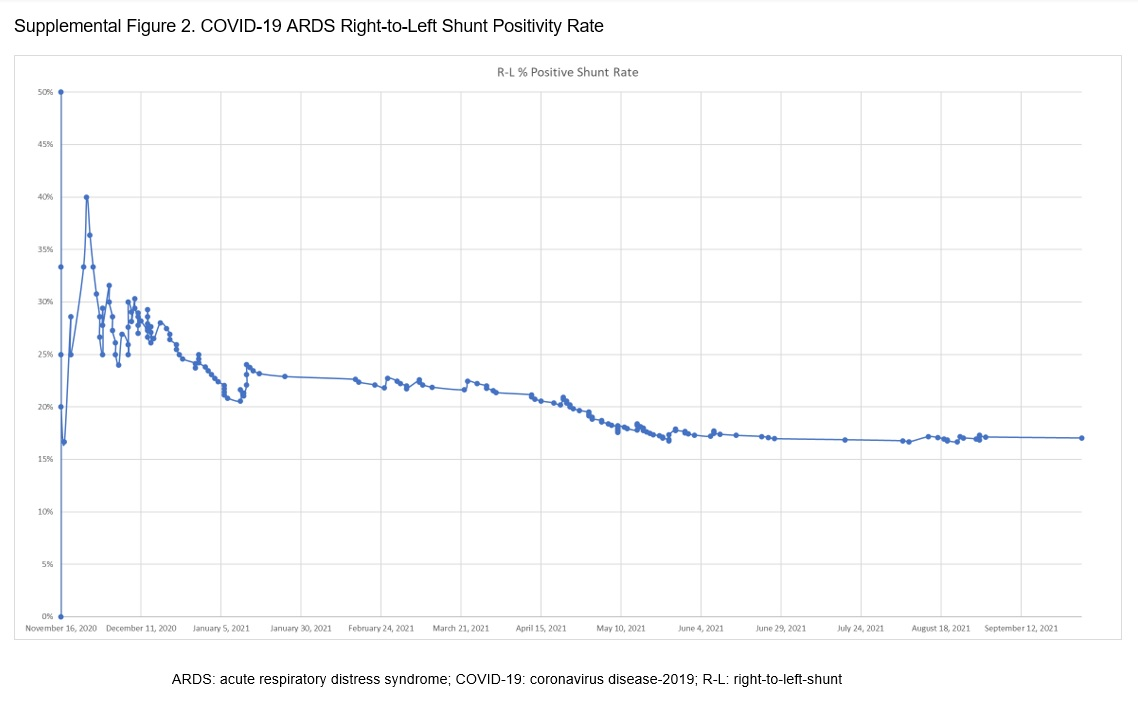

Supplement: Supplementary file 1 [file ccm-51-1023-s001.doc]
